# Serum COMP-C3b complexes in rheumatic diseases and relation to anti-TNF-α treatment

**DOI:** 10.1186/ar3694

**Published:** 2012-01-20

**Authors:** Kaisa E Happonen, Tore Saxne, Pierre Geborek, Maria Andersson, Anders A Bengtsson, Roger Hesselstrand, Dick Heinegård, Anna M Blom

**Affiliations:** 1 Department of Laboratory Medicine Malmö, Section of Medical Protein Chemistry, Lund University, Wallenberg Laboratory floor 4, SE-205 02 Malmö, Sweden; 2 Department of Clinical Sciences Lund, Section of Rheumatology, Lund University, Kioskgatan 3, SE-221 85 Lund, Sweden; 3 R and D Centre, Spenshult Hospital for Rheumatic Diseases, SE-313 92 Oskarström, Sweden; 4 Department of Clinical Sciences Lund, Section of Rheumatology, Lund University, BMC C12, SE-221 84 Lund, Sweden

## Abstract

**Introduction:**

Cartilage oligomeric matrix protein (COMP) is found at elevated concentrations in sera of patients with joint diseases such as rheumatoid arthritis (RA) and osteoarthritis (OA). We recently showed that COMP activates complement via the alternative pathway and that COMP-C3b complexes are present in sera of RA patients, but not in healthy controls. We now set out to elaborate on the information provided by this marker in a variety of diseases and larger patient cohorts.

**Methods:**

COMP-C3b levels in sera were measured by using an enzyme-linked immunosorbent assay (ELISA) capturing COMP and detecting C3b. Serum COMP was measured by using ELISA.

**Results:**

COMP-C3b levels were significantly elevated in patients with RA as well as in systemic lupus erythematosus (SLE), compared with healthy controls. SLE patients with arthritis had significantly higher COMP-C3b levels than did those without. COMP-C3b was furthermore elevated in patients with ankylosing spondylitis (AS), psoriatic arthritis (PsA), reactive arthritis, systemic sclerosis, and OA. COMP-C3b did not correlate with COMP in any of the patient groups. COMP-C3b correlated with disease activity in RA, but not in other diseases. COMP-C3b levels in RA patients decreased on treatment with tumor necrosis factor (TNF)-α inhibitors, whereas the levels increased in patients with AS or PsA. The changes of COMP-C3b did not parallel the changes of C-reactive protein (CRP).

**Conclusions:**

COMP-C3b levels are elevated in several rheumatologic diseases and correlate with inflammatory measures in RA. COMP-C3b levels in RA decrease during TNF-α inhibition differently from those of CRP, suggesting that formation of COMP-C3b relates to disease features not reflected by general inflammation measures.

## Introduction

Rheumatoid arthritis (RA) is a disabling disease with both a large impact on the quality of life for the patient and a high economic impact on society. It is known that early intervention minimizes tissue damage and disease progression. Therefore specific and sensitive diagnostics are essential for early discovery of disease. Even though rheumatoid factor (RF) and anti-citrullinated peptide antibodies (ACPAs) are widely used as diagnostics for RA, improvements are needed to enhance the specificity and sensitivity of current molecular markers in RA. Therapy should also improve when new diagnostic assays can differentiate RA patients into groups with different underlying pathologic mechanisms. Several approaches to develop novel serologic markers for RA have been attempted, one of them being measurement of cartilage oligomeric matrix protein (COMP) in serum or synovial fluid. COMP is a structural component of cartilage, and it has been shown to be released during erosive joint diseases such as RA and osteoarthritis (OA) [1]. Excessive production of COMP in the skin in systemic sclerosis (SSc) is also reflected by an increase in serum COMP [2]. COMP is a homopentamer of 435 kDa in which each individual monomer is composed of four epidermal growth factor (EGF) domains, eight thrombospondin type 3 (TSP3) domains, and a globular C-terminus. The chains polymerize via their N-terminal coiled coil domains, and this is stabilized by interchain disulfide bonds [3]. One function of COMP in tissue is to catalyze collagen fibrillogenesis [4,5] and, in the adult, to stabilize tissue structure by interacting with other collagen-bound matrix proteins [6].

In a previous study, we showed that COMP released from the joints during RA is able to activate complement both *in vitro *and *in vivo *[7]. Complexes between COMP and the complement-activation product C3b were found both in the serum and synovial fluid of RA patients, whereas no COMP-C3b was found in the serum of healthy controls or in patients with OA; therefore, it was concluded that COMP-C3b might be diagnostic in RA. The complement-activating site was shown to reside within the C-terminal proportion of COMP, by interaction with both properdin and C3.

Complement is an important part of innate immunity, and its uncontrolled activation has been strongly implicated in many autoimmune diseases, among them, RA. Several studies have shown that complement deficiency or inhibition ameliorates disease activity in rodent models of RA [8,9], and complement-activation products have been found in the joints of patients with RA [10-12]. Several proteins that are found in affected joints have been shown to activate complement, among them RF [13,14], ACPA [15], cartilage molecules of the small leucine-rich-repeat protein (SLRP) family [16,17], as well as apoptotic cells [18] and thereby most likely contribute to disease progression by feeding the inflammatory response. Measurement of complexes between complement-activation products and joint-specific molecules might provide improved specificity to the diagnostics of RA and may give better distinction between patients with complement-mediated joint inflammation and healthy individuals. This might also provide valuable information on the patients' need of complement-inhibition therapy that is currently undergoing clinical trials.

Our previous pilot study on the presence of COMP-C3b in the circulation of RA and OA patients, as well as in healthy controls, was conducted on a small number of patients, and therefore, in the present study, we set out to validate our results in larger patient cohorts. To investigate whether such complexes are a specific feature of RA or whether they can be found in patients with other rheumatologic disorders or joint diseases, we have now included patients with SSc, reactive arthritis (ReA), psoriatic arthritis (PsA), ankylosing spondylitis (AS), and systemic lupus erythematosus (SLE), as well as a larger group of healthy individuals. Furthermore, we have also related serum concentrations of COMP-C3b complexes to clinical and biochemical markers commonly used to monitor disease activity, as well as studied changes of COMP-C3b levels over a 3-month period in relation to treatment of RA, PsA, or AS with TNF inhibitors to elucidate how changes relate to modulation of disease activity.

## Materials and methods

### Patients and controls

The study was approved by the Ethical Review Board at Lund University. Table 1 describes some characteristics of the patients and controls. All serum samples were retrieved in a similar, standardized fashion (nonfasting) and were stored in -80°C after centrifugation. Samples from 98 RA patients were taken immediately before initiation of TNF-α inhibition. Of these, samples from 90 patients were also available after 6 and 12 weeks of treatment. All patients fulfilled the 1987 ACR criteria for RA [19]. Samples from 58 patients with symptomatic, radiographically verified knee OA with uni- or bilateral Kellgren-Lawrence grade 3 or more were retrieved before enrollment in a study of physical exercise [20]. Samples from 13 patients with ReA were obtained in connection with a knee-joint aspiration due to synovitis. Samples from 30 patients with PsA or AS were obtained immediately before initiation of TNF-α inhibition. Diagnosis of ReA, PsA, or AS was based on clinical judgment by the treating physician and included radiographic examinations when applicable. The patient charts of the ReA patients were examined to exclude alternative diagnoses. All AS patients had axial involvement but no clinical signs of peripheral arthritis, whereas the PsA patients had peripheral arthritis but no clinical signs of axial involvement. From the majority of these patients, samples were also available after 6 and 12 weeks of TNF-α inhibition.

**Table 1:** Description of patients and controls

	RA	OA	ReA	PsA	AS	SSc	SLE	Control
Number	98	58	13	30	30	40	56	97
Gender (F:M)	72:26	29:29	5:8	19:11	5:25	27:13	48:8	64:33
Age * (years)	55 (23-85)	56 (36-65)	29 (17-60)	48 (23-70)	41 (22-75)	54 (16-77)	42 (17-75)	45 (23-74)
Disease duration (years)	7.9 (0.8-39)	Not available	0.04 (0-0.19)	6.3 (1.7-23.3)	15.6 (0.2-55.2)	1.5 (0.5-19)	5.0 (0.35)	Not applicable
COMP-C3b (AU)	0.42 (0.19-1.19)	0.58 (0.27-1.36)	0.74 (0.38-1.14)	0.31 (0.19-0.92)	0.33 (0.18-0.51)	0.76 (0.15-1.43)	0.50 (0.27-0.85)	0.20 (0.09-0.83)
COMP (U/L)	9.1 (3.3-24.4)	11.2 (6.4-22.1)	8.9 (4.4-11.2)	8.2 (4.1-15.3)	6.8 (3.4-12.4)	10.5 (2.3-29.0)	5.6 (3.0-13.3)	7.0 (3.2-12.1)
CRP (mg/L)	15.5 (0-126)	< 5 (< 5-15)	Not determined	9.3 (1-205)	7.9 (0-65)	12 (0.6-91)	Not determined	Not determined
ESR (mm/h)	29 (2-110)	6 (1-35)	48 (2-100)	18 (4-95)	14 (2-64)	18 (2-90)	Not determined	Not determined
TNF-α inhibitor infliximab/etanercept/adalimumab	98/0/0			22/6/2	9/15/6			

*Values denote median and range, except for gender and tumor necrosis factor (TNF)-α inhibitor. AS, ankylosing spondylitis; PsA, psoriatic arthritis; RA, rheumatoid arthritis; OA, osteoarthritis; ReA, reactive arthritis; SLE, systemic lupus erythematosus SSc, systemic sclerosis.

The 40 patients with SSc all fulfilled the ACR criteria for SSc [21]. The disease was classified as diffuse cutaneous SSc (dcSSc; *n *= 30) or limited cutaneous SSc (lcSSc; *n *= 10) according to the extent of skin involvement [22]. Skin involvement was determined by the modified Rodnan skin score (mRSS) [23]. The disease onset was defined as the first non-Raynaud manifestation. The 56 SLE patients included in the study fulfilled four or more ACR classification criteria [24]. Disease activity was evaluated by using SLEDAI-2K (SLE disease activity index) [25]. Thirty patients had no clinical disease activity and were in remission. Twenty-six patients had clinical disease activity, defined as a flare, at the time of blood sampling. The SLEDAI score at this time varied between 2 and 20. Routine laboratory tests were used to assess disease activity, including concentration of complement components (C3, C4, and C1q) as well as presence of antibodies against dsDNA, C1q, SS-A, and SS-B. Ninety-seven healthy volunteers with no history of rheumatologic disease were selected for the study as a control group. Sera from these individuals were collected in Lund and in Malmö, according to standard procedures, as described for the patients. Informed consent was obtained from all participants involved in the study.

### Measuring COMP and COMP-C3b complexes in serum

Serum COMP concentrations were measured with a commercially available COMP ELISA (AnaMar, Lund, Sweden). The presence of soluble COMP-C3b complexes in biologic samples was measured with a sandwich-ELISA based on the COMP ELISA plate. Serum was diluted 1:10 in the sample buffer provided with the kit, and a 50-μl sample was added to the provided anti-COMP-coated plates. As an internal reference sample, 50 μl of the 1.7 U/L calibrator diluted 1:2 in sample buffer was added to duplicate wells on the plate. Incubation was at room temperature for 2 hours, after which plates were washed 4 times with the washing buffer provided in the kit. A biotinylated polyclonal anti-C3d antibody (A0063; Dako) diluted 1:1,000 in the conjugate buffer of the kit, was added to the wells, and the plates were incubated for 1 hour at room temperature. After washing as described earlier, HRP-conjugated streptavidin (21130; Pierce) was diluted 1:60,000 in the conjugate buffer and incubated with the plate for 1 hour at room temperature. After washing 4 times, bound complexes were detected according to the protocol of the COMP ELISA. The recorded absorbance of each sample was normalized by setting the mean absorbance of the internal reference to 1 (450 nm; Cary 50 MPR microplate reader, Varian). All samples were measured in duplicate, and values are expressed as arbitrary units (AUs).

The presence of rheumatoid factor does not interfere with the assay because a biotinylated primary antibody in combination with a streptavidin/HRP-conjugate is used to avoid secondary antibodies. Therefore, any rheumatoid factors present that might recognize the murine-catching antibody will not generate signal in the detection step. In support of this, we found no correlation between the presence of rheumatoid factor and level of COMP-C3b in sera.

### Biotinylation of antibodies

The polyclonal anti-C3d antibody (A0063; Dako) was biotinylated by using biotin-amidohexanoic acid-*N*-hydroxysuccimide ester (B2643; Sigma). In brief, the antibody was incubated with biotin-amidohexanoic acid-*N*-hydroxysuccimide ester in 0.1 *M *borate buffer, pH 8.0, overnight at +4°C at a ratio of 0.5 mg biotinylation reagent per 1 mg antibody. The reaction was stopped by adding solid glycine to the mixture to a final concentration of 4 mg/ml. The antibody was dialyzed extensively against TBS, and successful biotinylation was confirmed by subjecting the sample to Western blot with a streptavidin-HRP conjugate (21130; Pierce).

### Statistical analysis

The differences in COMP and COMP-C3b concentrations between disease groups were analyzed by using a Kruskal-Wallis test and a Dunn Multiple Comparison posttest. Two-group comparisons were conducted by using a Mann-Whitney *U *test. The Friedman test was used to calculate statistically significant changes in inflammatory parameters on TNF-α inhibition in the RA patients. Because of missing values for both the AS and PsA patients at 6 weeks and 3 months, we used a Wilcoxon matched-pairs test to include more observations in the analysis of these groups. Two-parameter correlations were conducted with Spearman correlation analysis.

## Results

### Elevated COMP-C3b is found in several rheumatologic diseases

**Figure 1:**
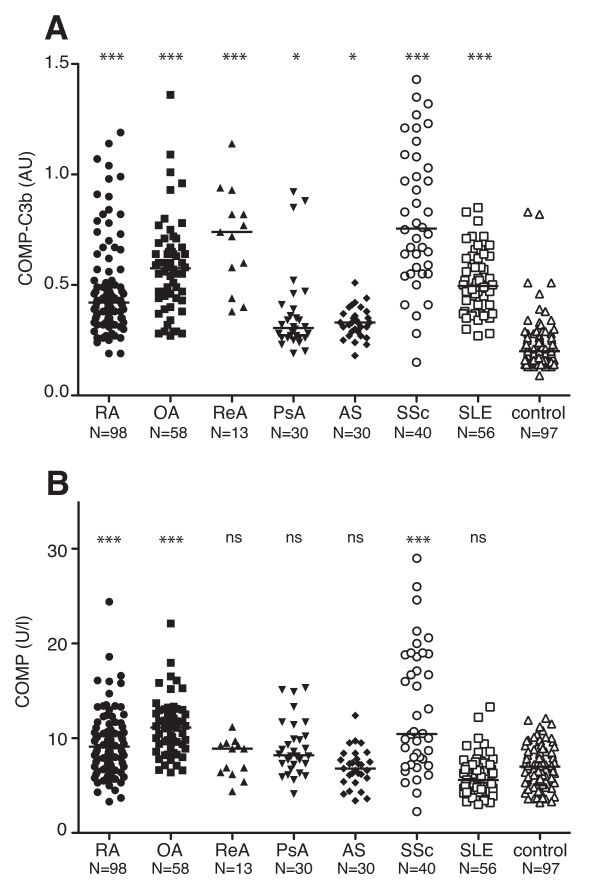
**Serum COMP-C3b and COMP in patients with rheumatologic diseases**. Serum COMP-C3b **(a) **and COMP **(b) **were measured in patients with different rheumatologic diseases. The horizontal bar indicates the median of each group. Statistical significance of differences between disease groups and controls was measured by using a Kruskal-Wallis test with a Dunn multiple comparison posttest. ***P < 0.001; **P < 0.01; *P < 0.05; ns, not significant.

COMP-C3b concentrations were significantly elevated in the serum of RA patients compared with healthy controls (*P *< 0.001), corroborating and extending previously published results (Figure 1a and Table 1) [7]. In contrast to earlier findings, the OA patients in this study cohort displayed high COMP-C3b concentrations in their sera (*P *< 0.001), although, as for RA patients, the scatter between the individuals was relatively large. High COMP-C3b concentrations were also found in the sera of patients with SSc, ReA, and SLE (*P *< 0.001). Patients with PsA and AS had moderately elevated levels of serum COMP-C3b (*P *< 0.05). Applying a cut-off value represented by the median + 2SD from the control group, 43.9% of RA, 77.6% of OA, 84.6% of ReA, 20% of PsA, 6.7% of AS, 67.9% of SLE, 87.5% of SSc patients, and 5.2% of healthy controls tested positive for COMP-C3b.

Elevated COMP levels were found only in patients with RA, SSc, and OA (*P *< 0.001), compared with healthy controls (Figure 1b and Table 1). No correlation was found between serum COMP and COMP-C3b in any of the patient groups. No difference in COMP-C3b values were observed between male and female subjects in any of the disease groups.

### COMP-C3b correlates with disease activity in RA

COMP-C3b was found to correlate to several measures reflecting disease activity in RA. A positive correlation was found between COMP-C3b and the disease-activity score (DAS28-ESR or CRP-based) (Table 2). Furthermore, COMP-C3b correlated with the individual items of DAS28 (that is, C-reactive protein (CRP), the 28 swollen and tender joint counts, and the erythrocyte sedimentation rate (ESR)). The fact that these correlations are relatively weak suggests that even though the serum concentration of COMP-C3b relates to disease activity, it reflects features of the disease process not measured by traditional inflammatory variables. No correlation was found between COMP-C3b and the visual analogue scale (VAS) global assessment score or pain rated on VAS in RA patients.

**Table 2:** Correlation of COMP-C3b with clinical variables in RA

	Spearman *r*	*P *value
CRP	0.3551	0.0004
DAS28-ESR	0.3770	0.0002
DAS28-CRP	0.2888	0.0043
CDAI	0.2563	0.0109
SDAI	0.2779	0.0061
28 swollen joints	0.2109	0.0371
28 tender joints	0.2313	0.0220
ESR	0.3556	0.0004
Hb	-0.2289	0.0256
VAS global	0.0863	0.3983
VAS pain	0.1413	0.1652

CDAI, clinical disease activity index; CRP, C-reactive protein; DAS, disease activity score; ESR, erythrocyte sedimentation rate; Hb, hemoglobin concentration; SDAI, simplified disease activity index; VAS, visual analogue scale.

Interestingly, but not unexpectedly, COMP-C3b did not correlate with the DAS28 in patients with PsA (*r*_s _= 0.3106; *P *= 0.0948) or AS (*r*_s _= 0.2400; *P *= 0.2015). No correlation was found between COMP-C3b and the VAS global-assessment score or pain rated on VAS in AS (global-assessment score; *r*_s _= -0.0956; *P *= 0.6152; pain; *r*_s _= 0.1064; *p *= 0.5758) or PsA (global-assessment score; *r*_s _= 0.0084; *P *= 0.9650; pain; *r*_s _= 0.2326; *P *= 0.2162). Moreover, no correlation was found between CRP and COMP-C3b in these disease groups (PsA, *r*_s _= 0.2672; *P *= 0.1534; and AS, *r*_s _= 0.1198; *P *= 0.5358). Therefore it seems that circulating COMP-C3b is not closely linked to disease activity in these patient groups. No significant correlation was found between COMP-C3b and ESR in ReA (*r*_s _= 0.54555; *P *= 0.0876), possibly because of the relatively few patients in this group. However, in SSc patients, a weak correlation was found between COMP-C3b and CRP (*r*_s _= 0.5246; *P *= 0.0306). COMP-C3b did not correlate with the ESR (*r*_s _= 0.146; *P *= 0.312) in OA patients.

### COMP-C3b in SLE

**Figure 2:**
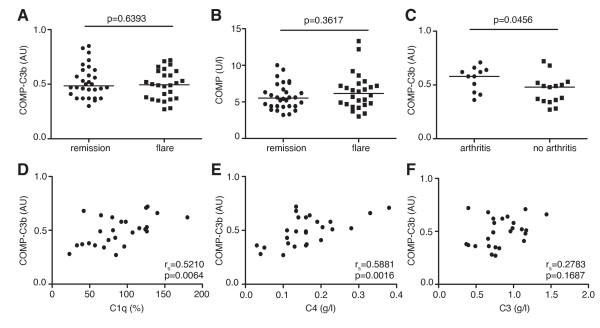
**COMP-C3b in SLE patients**. Comparison of serum COMP-C3b **(a) **and COMP **(b) **in systemic lupus erythematosus (SLE) patients during remission (*n *= 30) and flare (*n *= 26). SLE patients with arthritis during flare (*n *= 11) have higher COMP-C3b than do patients without arthritis during flare (*n *= 15) **(c)**. Correlation between COMP-C3b and C1q **(d)**, C4 **(e)**, C3 **(f) **in SLE patients during flare was measured by using the Spearman correlation analysis.

The levels of COMP-C3b in SLE patients were increased compared with healthy controls, but no difference was noted in serum COMP-C3b or COMP between SLE patients in flare or in remission (Figure 2a, b). However, SLE patients with arthritis as a part of the disease flare at the time of sampling had significantly higher serum COMP-C3b concentrations than did patients without arthritis (Figure 2c). Patients who at any time of the disease course had had arthritis showed, in general, slightly higher COMP-C3b values in their sera compared with patients that never had arthritis, although this did not reach statistical significance. No correlations were apparent for any other SLE disease phenotypes (such as, for example, nephritis, when investigating the ACR criteria for SLE (data not shown). During disease flare in SLE, a strong positive correlation occurred between COMP-C3b and complement components C1q and C4, but no correlation between COMP-C3b and C3 (Figure 2d through f). Even though SLE patients displayed relatively low COMP concentrations in serum, they had significantly elevated amounts of COMP-C3b in the circulation, comparable to those in RA patients (Figure 1a, b). This may suggest that the fragments of COMP released in SLE particularly represent those that can activate complement. This is in contrast to other studied disease groups in which the ratio between circulating COMP-C3b and COMP was much lower; the only interesting exception was patients with ReA who also had relatively high levels of serum COMP-C3b compared with COMP.

### COMP-C3b decreases in RA patients with TNF-α inhibition

**Figure 3:**
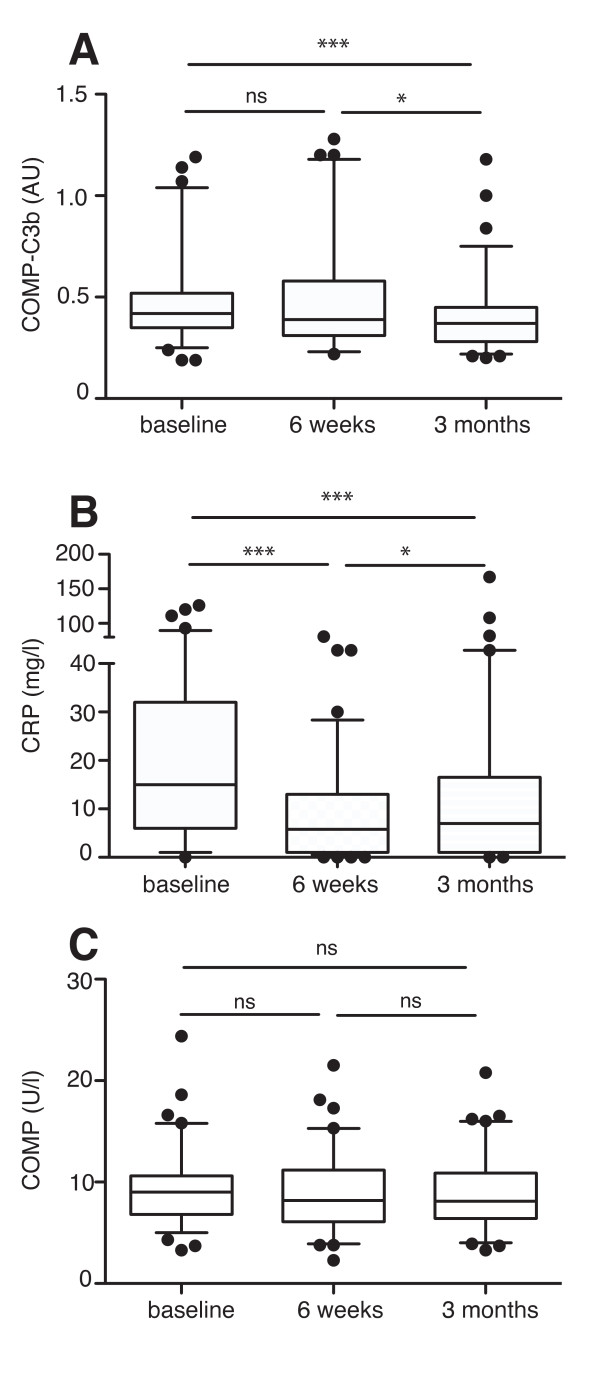
**COMP-C3b decreases in rheumatoid arthritis (RA) patients on tumor necrosis factor (TNF)-α inhibition**. RA patients (*n *= 90) receiving infliximab were measured for COMP-C3b **(a)**, C-reactive protein (CRP) **(b)**, and COMP **(c) **at baseline, after 6 weeks, and after 3 months of treatment. Statistical significance of changes in these parameters was measured with a Friedman test. ***P < 0.001; **P < 0.01; *P < 0.05; ns, not significant.

TNF-α inhibition has been shown to reduce serum COMP levels in patients with RA [26]. As we showed that COMP-C3b reflects disease activity in RA patients, we hypothesized that TNF-α inhibition might also reduce the serum COMP-C3b concentrations. Samples collected from 90 RA patients were measured for serum COMP-C3b, COMP, and CRP at baseline, after 6 weeks, or after 3 months of infliximab treatment. No significant difference was noted between baseline COMP-C3b and the 6-week values, whereas COMP-C3b was significantly reduced after 3 months of treatment (Figure 3a). Notably, the decrease in COMP-C3b was marked in some patients, whereas the levels remained unchanged in others. CRP levels were reduced in the majority of patients already after 6 weeks of treatment compared with baseline and remained low at 3 months (Figure 3b). Therefore, it seems that the decreases in CRP levels and COMP-C3b follow different kinetics, with reduction in COMP-C3b being observed at a later stage. However, COMP-C3b still correlated with CRP at 6 weeks (*r*_s _= 0.3531; *P *= 0.001) and 3 months (*r*_s _= 0.388; *P *= 0.001). Serum COMP values remained unchanged in these patients even at 3 months, for reasons still not known. One possible explanation is the relatively low baseline COMP values in this cohort (Figure 3c).

### COMP-C3b increases in PsA and AS patients with TNF-α inhibition

**Figure 4:**
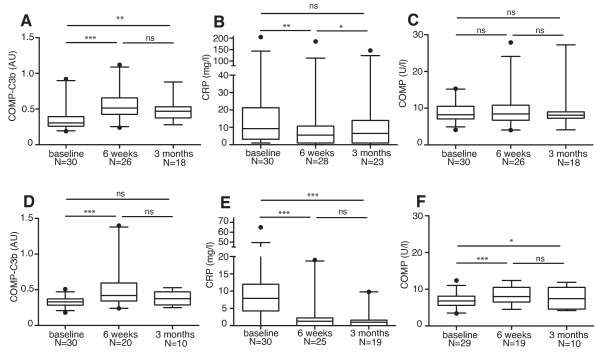
**Serum COMP-C3b increases in patients with ankylosing spondylitis (AS) and PsA on tumor necrosis factor (TNF)-α inhibition**. Serum COMP-C3b **(a)**, C-reactive protein (CRP) **(b)**, and COMP **(c) **were measured in patients with PsA receiving TNF-α inhibition at baseline, after 6 weeks of treatment, and after 3 months of treatment. COMP-C3b **(d)**, CRP **(e)**, and COMP **(f) **were also measured in AS patients receiving TNF-α inhibition at the same times. Statistical significance of changes in these parameters was measured with a Wilcoxon matched-pairs test because of many missing values at the time point, 3 months. ***P < 0.001; **P < 0.01; *P < 0.05; ns, not significant.

Interestingly, in PsA patients receiving TNF-α inhibition therapy, serum COMP-C3b was increased after 6 weeks compared with baseline and remained elevated at 3 months of treatment (Figure 4a). CRP levels in these patients decreased as expected after 6 weeks of treatment compared with baseline, and a slight increase was observed again at 3 months (Figure 4b). A similar pattern was seen for AS patients receiving TNF-α inhibition therapy; COMP-C3b increased at week 6 compared with baseline, and a statistically insignificant decrease was observed at 3 months (Figure 4d). In line with the findings for PsA patients, in AS, the CRP levels had already stabilized at a low level by week 6 and remained low at 3 months (Figure 4e). Serum COMP levels remained unchanged in the PsA group during treatment, whereas they increased in the AS group (Figure 4c and 4f, respectively). Taken together, this suggests that COMP-C3b does not closely relate to ongoing inflammation in these diseases.

## Discussion

In the present study, we showed that elevated COMP-C3b can be found in the circulation of RA patients, extending previous observations in a smaller patient cohort. COMP-C3b correlates with several inflammatory parameters describing mainly synovitis in RA, suggesting that joint inflammation causes the release of COMP fragments that are able to activate complement. Interestingly, with TNF-α inhibition, serum COMP-C3b decreased significantly, which indicates that the molecular process of cartilage turnover has been altered. Because serum COMP did not significantly decrease during TNF-α inhibition, it appears that the intervention causes a reduction in the release of complement-activating COMP, perhaps in the form of unique fragments, as opposed to a reduction in total COMP. This might be a process of modified cartilage degradation or turnover due to an alteration of protease activity. A previous study reported that serum COMP levels decreased on TNF-α inhibition [26]. The fact that this decrease was not seen in our patient cohort might be a reflection of the lower baseline COMP values in our cohort (9.1 U/L) compared with the other cohort (11.3 U/L and 12.2 U/L in the different treatment groups). Furthermore, in the other study, the major reduction was seen in the patients with the highest baseline COMP values.

The reduction in CRP levels and COMP-C3b were observed to follow different kinetics, with a decrease in COMP-C3b occurring at a slower rate. This indicates that formation of COMP-C3b is not an immediate response to inflammation-induced joint damage but rather a complicated process regulated by cartilage metabolic and catabolic activity and by the surrounding microenvironment.

The AS patients in our cohort, who were chosen for having only axial joint involvement, had slightly higher concentrations of circulating COMP-C3b than did the controls. Interestingly, in this group, CRP did not correlate with COMP-C3b. Similar observations were made for patients with peripheral joint PsA, in which a lack of correlation between CRP and COMP-C3b was also observed. The fact that both groups had similar levels of circulating complexes indicates that the origin of released COMP fragments (axial/peripheral joints) does not directly relate to the ability of COMP to activate C3. Furthermore, lack of correlation between CRP and these complexes shows that the level of COMP-C3b in these patient groups does not reflect disease activity. Surprisingly, an increased COMP-C3b was observed in both AS and PsA patients on TNF-α inhibition, even though systemic inflammation was reduced, as shown by a decrease in CRP. Therefore, it is possible that this intervention either stimulates tissue regeneration or changes the character of the COMP fragments released from the tissue. It also is possible that TNF-α inhibition is effective downstream of the process of tissue destruction and complement activation and therefore will primarily have an effect on the inflammation, its symptoms, and effects.

In the current study, we observed elevated levels of COMP-C3b in the sera of OA patients, in contrast to our previous report, in which this was observed only in the synovial fluid and not in serum. With further investigations of this divergence, we found that, for unexplained reasons, in the serum of OA samples previously used, all C3 was degraded to fragments smaller than 40 kDa, as shown by SDS-PAGE followed by Western blotting. Thus, the fraction of C3b that, in the patient, was deposited onto COMP was most likely similarly degraded and therefore unrecognizable in our assay. We found that this was not the case for the synovial fluid samples. We are not sure what caused the degradation of C3 in the archival OA samples during storage. We have found experimentally that serum can be thawed and frozen several times or stored for many hours at room temperature before freezing without C3 becoming degraded, and thus no extreme precautions must be taken for the sample to be suitable for analysis with our assay. However, the OA serum samples used in the previous study were obviously exposed to some harsh conditions that were not apparent to any co-authors but could have occurred during the many years during which these samples were stored. We confirmed that C3 was not degraded in any other patient group or in OA samples used in the current study. The new data clearly show that at least a proportion of the COMP released in OA contains the complement-activating region in the C-terminal part. It is important to stress that, even though the inflammation is much more pronounced in RA, an inflammatory component also exists in OA [27,28].

Patients with SSc displayed in previous studies elevated COMP levels in their circulation as a result of increased COMP synthesis and turnover by their dermal fibroblasts [2]. We could now observe elevated levels of COMP-C3b in their circulation as well, which shows that the released COMP fragments have the potential to activate complement. Furthermore, patients with ReA displayed high levels of COMP-C3b in their circulation. The median COMP-C3b values in ReA were markedly higher than those in other studied disease groups, which most likely reflects the pronounced cartilage engagement and strong inflammatory stimulation of the disease. Similarly, SLE patients were found to have elevated levels of COMP-C3b in their circulation, independent of being in disease flare or remission. The serum COMP-C3b concentration was, however, significantly higher in patients with arthritis than in patients without arthritis, suggesting the importance of joint inflammation and possibly cartilage involvement for the release of complement-activating COMP. The fact that C1q and C4 levels were positively correlated with COMP-C3b shows that classic pathway components are not consumed during the disease process leading to COMP-C3b formation. Even though a negative correlation could be expected between COMP-C3b and C3, it might be that the overwhelming excess of C3 in serum compared with released COMP masks such changes. However, we cannot exclude that C3 has been consumed within the synovial fluid as a more-local response.

## Conclusions

We found elevated levels of COMP-C3b complexes in the circulation of patients with several rheumatologic diseases, showing that the release of complement-activating COMP is not a specific feature of RA. Because levels of COMP-C3b correlate to the inflammatory variables in RA but not in the other diseases examined, it seems that formation and release of complexes can reflect somewhat different processes in different conditions. This may relate to downstream regulation of the inflammatory process and merits further investigation. Notably, TNF-α inhibition in RA reduced COMP-C3b serum levels in RA, but not in AS and PsA, which points to different pathophysiologic mechanisms regulating complex formation. It should be stressed that although a correlation was found to inflammation in RA, this was rather weak and, taken together with the different kinetics of response for CRP and COMP-C3b complexes after TNF-α inhibition, may suggest that the release of complexes is not merely due to inflammation but reflects other, yet unknown components of the disease process in RA (for example, being upstream of major cytokine release). Further studies, including longitudinal monitoring of early RA patients, are needed to elucidate the pathophysiologic role of COMP-C3b complexes and to establish their value as disease markers in the clinic.

## Abbreviations

ACPA: anti-citrullinated peptide antibody; AS: ankylosing spondylitis; COMP: cartilage oligomeric matrix protein; CRP: C-reactive protein; DAS: disease activity score; EGF: epidermal growth factor; ESR: erythrocyte sedimentation rate; OA: osteoarthritis; PsA: psoriatic arthritis; RA: rheumatoid arthritis; ReA: reactive arthritis; RF: rheumatoid factor; SLE: systemic lupus erythematosus; SLEDAI: SLE Disease Activity Index; SLRP: small leucine-rich repeat protein; SSc: systemic sclerosis; TNF-α: tumor necrosis factor-α; VAS: visual analogue scale.

## Conflicting interests

The authors KEH, AMB, TS, and DH have filed a patent application on a method to detect tissue degradation leading to inflammation. Authors TS and DH own stocks in AnaMar Medical, who provided the COMP-kits for the study.

## Authors' contributions

KH carried out the COMP-C3b ELISAs, did the statistical analysis, and drafted the manuscript. TS provided patient material, participated in the study design, and helped draft the manuscript. PG participated in statistical analysis of the data. MA, AAB, and RH provided patient material and revised the manuscript. DH participated in the study design and helped draft the manuscript. AB participated in the study design, helped draft the manuscript, and supervised the study. All authors read and approved the manuscript.
